# The effect of free school fruit on academic performance: a nationwide quasi-experiment

**DOI:** 10.1038/s41598-023-48095-4

**Published:** 2023-11-27

**Authors:** Torleif Halkjelsvik, Elling Bere

**Affiliations:** 1 Centre for Evaluation of Public Health Measures, Norwegian Institute of Public Health, Folkehelseinstituttet, Skøyen, Postboks 222, 0213 Oslo, Norway; 2 Department of Alcohol, Tobacco and Drugs, Norwegian Institute of Public Health, Oslo, Norway; 3 Department of Health and Inequalities, Norwegian Institute of Public Health, Oslo, Norway; 4 Faculty of Health and Sport Sciences, University of Agder, Kristiansand, Norway

**Keywords:** Psychology, Risk factors

## Abstract

In past research, higher intake of fruit has been associated with better academic achievement. In Norway, the government required lower secondary schools to provide fruit to their pupils from 2007 to 2014. The present study assessed whether this policy improved academic performance. In addition to secondary schools, the policy covered schools with combined elementary and lower secondary education, but not ordinary elementary schools. This differentiation, in combination with administrative data on test scores before, during, and after the law was enforced, created a nationwide quasi-experiment. Population register data on parents’ sociodemographic characteristics allowed for targeted analyses on a subsample of boys with low sociodemographic status. In analyses of 5th grade tests, the free fruit policy coincided with a slight decline in test scores among eligible compared to non-eligible pupils in the subsample (B = − 0.18, 95%CI[− 0.35, − 0.01]) and entire population (B = − 0.14, 95%CI[− 0.24, − 0.05]). Exploratory analyses of exam data in 10th grade yielded similar results, and sensitivity tests either failed to detect any effect or demonstrated a negative tendency. In a Western country with low levels of food insecurity, a policy that required schools to provide free fruit to pupils did not appear to improve academic performance.

## Introduction

The school provides a convenient arena for interventions and measures targeting childhood nutrition. In developed countries, universal free school meals^1^ and improved school meal quality^2^ have been reported to improve academic performance. Long-term beneficial outcomes such as higher educational attainment and higher adult income have been reported as potential effects of the current Swedish school meal program^3^, the historical Norwegian Oslo breakfast^4^, and the US National School Lunch Program^5^. The literature on healthy eating at school and academic performance has mainly focused on broad nutritional interventions relating to meals such as lunch and breakfast, but to our knowledge, no studies have systematically assessed the impact of specific types of food, such as fruits.

In general, fruit is considered an important component of recommended healthy diets^6^, and it may contribute to preventing a range of chronic diseases^7^ that can hamper social and cognitive functioning. Fruit contains several basic nutrients, as well as other compounds with potential benefits beyond basic nutrition. Particular nutrients and secondary metabolites found in fruits act on molecular systems and cellular processes that are vital for maintaining cognitive function^8,9^, also for young people^10^. In research on fruits, polyphenols^11,12^ have gained great attention. Flavonoids (a polyphenol, abundant in fruits) might benefit cognitive outcomes within an acute time frame of 0–6 h^13^. Gut microbiota is considered important for cognition^14^, and eating fruit contributes to a healthy gut microbiota^15^. Youth also consume many ultra-processed foods that might be negatively linked to cognition and learning^16^, and fruit may replace such unhealthy food^17^.

Fruit intake has been associated with better cognitive performance^18^. It might increase concentration in school children^19^, which may reduce negative behaviors (off-task, out-of-seat) that impair the learning environment for the other children in the classroom. In several international studies, fruit intake and diet quality in general have been associated with better academic performance^20–22^. This has also been shown in Norway. A recent study of 15–17-year-olds reported that among girls, 40% of those with high academic achievement ate fruit daily, while the figure was only 25% for those with low academic achievement^23^.

As previous studies of fruit and academic performance are observational and often cross-sectional, causality can not be established. Potential for confounding is great. For example, there is a social gradient in health and health behaviors, and also in fruit intake in adolescents in Norway^24^. Healthy eating is correlated with other behaviors that themselves might affect academic performance, such as physical activity and healthy sleep habits^25^. Furthermore, intelligence, which is one of the main determinants of academic performance, might also be related to dietary choice and fruit intake^26^.

Such problems of confounding represent a key problem in nutrition epidemiology, and fewer but larger (mega) trials have been suggested as a solution to achieve better answers to the most important questions^27^. However, such trials are difficult to conduct^28^, and therefore rare. Due to the nature of the implementation of a nation-wide free school fruit policy in Norway^29^, the current study circumvents some of the methodological problems of past research on fruit and academic performance.

In 2007, the Norwegian Government mandated that all pupils attending schools with lower secondary education, encompassing grades 8–10 and ages 13–16, receive a piece of fruit every school day at no cost. In 2008 this was required by law after a proposal from the Ministry of Education^30^. The law was repealed in 2014^31^. The policy included all schools with lower secondary education, which also meant that elementary pupils in combined elementary and lower secondary schools (20% of elementary pupils in Norway) were covered. Children in ordinary elementary schools (80%) were not eligible for free fruit. This allows for a comparison of the development of the academic performance of pupils eligible and not eligible for free fruit during elementary school.

The consumption of fruits in Norway has traditionally been low, and before the free school fruit implementation, it was argued that Norwegians ate less than half of what is recommended by the government^32–34^. According to earlier reports, the free fruit policy increased average fruit intake by approximately half a portion (about 30%) among the children attending schools covered by the policy^35,36^, and it increased the proportion eating fruits daily by 10 percentage points (from 57 to 67%)^37^. Furthermore, it has been reported that it reduced unhealthy snack consumption in children from families with low socioeconomic status^17^.

The government’s argument for providing free fruit to school children was that a healthy diet would contribute to better learning^30,38^. While this aligns with some of the existing research linking fruit consumption to cognitive benefits and academic performance^8,9,18–23^, the policy's impact on academic outcomes has not been empirically tested.

Therefore, the aim of this study is to assess whether the free fruit policy led to improvements in academic performance. We were particularly interested in pupils with potentially low intake of fruit and lower-than-average performance. Boys and pupils of lower socioeconomic status are found to eat less fruit than girls and pupils of higher socioeconomic status^39^ and perform worse in school^40^. In addition to the targeted analyses of this subsample, we assessed the potential impact of the policy on the academic performance of all registered pupils in Norway.

## Results

### Descriptive results

Pupils who were eligible for the free fruit policy serve as the treatment group in this study, and pupils who were non-eligible serve as the control group. Table 1 presents the demographic characteristics of the control and treatment groups for analyses of 5th grade test scores.

**Table 1:** Descriptive statistics for observations in control and treatment schools, 5th grade tests.

	Control	Treatment	Undetermined
	*N*	*N*	*N*
Years	13	13	13
Schools	1920	791	33
School-years	20,936	8649	71
Pupils	625,454	163,409	1580
Observations	1,808,163	469,862	4440
Missing test score	94,608	30,383	316
Non-standard test	41	20	2
Valid standard tests	1,713,517	439,461	4122

	%	%	%
Female	49.5	49.5	50.5
Non-immigrant	76.8	76.1	71.0
Higher education, mother	50.8	47.1	56.9
Higher education, father	38.5	32.3	45.0

	Mean (SD)	Mean (SD)	Mean (SD)
Mother’s income^a^	348’ (260’)	320’ (238’)	212’ (297’)
Father’s income^a^	557’ (479’)	495’ (400’)	596’ (773’)
Centrality^b^	834 (111)	760 (163)	808 (125)
Test scores (all years)			
Mathematics	25.8 (9.5)	24.0 (9.6)	24.6 (9.7)
English	25.7 (9.5)	24.4 (9.8)	27.1 (9.9)
Reading	19.2 (6.4)	18.3 (6.5)	18.6 (6.3)
Standardized scores			
Mathematics	0.04 (1.00)	− 0.15 (1.00)	− 0.10 (1.02)
English	0.03 (0.99)	− 0.11 (1.02)	0.22 (1.10)
Reading	0.03 (0.99)	− 0.12 (1.02)	− 0.06 (1.00)

Note. ^a^Income is reported in NOK 1000, and negative values are trimmed to 0.

^b^School-level variable indicating urbanicity of the municipality, maximum = 1000. Test scores range from 0 to 51.

**Figure 1:**
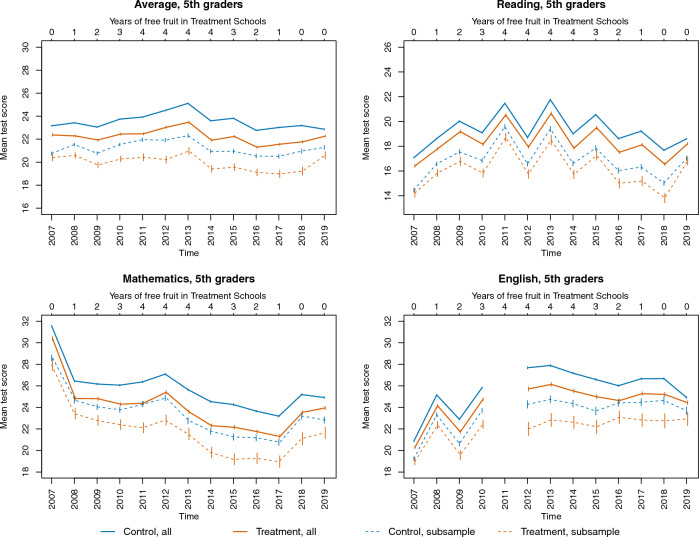
Averages of original scores for all 5th graders and subsample of boys with low socioeconomic status. Error bars are 95% confidence intervals of means. Upper horizontal axes indicate the number of years the pupils had received free fruit (degree of exposure) in the treatment schools.

The developments in test scores from 2007 to 2019 are presented in Fig. 1. The data include all registered scores of the three 5th grade tests for boys with low socioeconomic status (dashed lines) and the full sample of all 5th graders in Norway (solid lines). The first panel displays test scores averaged over the subjects Mathematics, English, and Reading, while the subsequent panels show the average scores for each subject separately. The scores are displayed on their original scales (for scores standardized per year, see Supplemental Materials, Figs. S1 and S2).

In Fig. 1, the number of years the pupils in the treatment schools received free fruit is indicated in the upper horizontal axes, labelled “Year of free fruit in Treatment Schools” (ranging from 0 to 4 years). Years of free fruit due to the policy in the control group are consistently zero and are not indicated in the figure. As observed from the graphs in Fig. 1, the scores in control and treatment schools develop in parallel, but there appears to be a slightly larger discrepancy during the years of receiving free fruit.

### Main analyses

Results from pre-registered regression analyses are presented in Table 1. The Phase-in term represents free fruit exposure when the policy was in effect, whereas the Phase-out term is exposure after the policy was abolished (free fruit during elementary school but not the most recent year or years). As stated in the pre-registration, we focus on the former. Relative to the development in the control group, a one-year increase in the exposure to the free fruit policy was associated with a decrease in test scores of 0.18 in the subsample of boys with low socio-economic status (see Table 1, upper panel under “Number of ‘fruit-years’”). In the full sample of all 5th graders (Table 1, lower panel under “Number of ‘fruit-years’”), the reduction in scores relative to the control group was estimated as 0.14 per year of free fruit. Results for analyses of exposure to any number of years of free fruit (a dichotomous variable) are presented under the last columns of Table 1 (“Any fruit”). Any exposure, which gives a weighted average of all levels of exposure, was associated with a decrease in test scores of 0.66 points for the subsample and 0.50 for the full sample. The direction of the estimated effect was contrary to our expectations.

The coefficients for the Phase-out, which measures exposure when the policy was no longer active (exposure to free fruit but not the most recent year), were also negative, but of lower magnitude and high statistical uncertainty. Similar results as presented in Table 2 were obtained also for alternative specifications of the impact model (see Supplementary Materials, Table S1).

**Table 2:** Regression coefficients (B), confidence intervals (CI), and p-values (p) for analyses of boys of low socio-economic status (subsample) and all 5th graders (full sample).

	Number of “fruit-years”			Any fruit		
	*B*	[95% CI]	*p*	*B*	[95% CI]	*p*
Subsample						
Phase-in	− 0.18	[− 0.35, − 0.01]	0.04	− 0.66	[− 1.44, 0.13]	0.10
Phase-out	− 0.07	[− 0.39, 0.24]	0.62	− 0.22	[− 1.01, 0.58]	0.53
Full sample						
Phase-in	− 0.14	[− 0.24, − 0.05]	0.004	− 0.50	[− 0.99, − 0.02]	0.05
Phase-out	− 0.15	[− 0.42, 0.12]	0.26	− 0.36	[− 1.02, 0.31]	0.24

Note. Controlled for eligibility of fruit at the time of testing. Analysis of Subsample (n = 203,142) included fixed effects of school by test type (n = 7388) and year (n = 13); Analysis of Full Sample (n = 2,152,909) included fixed effects of school by test type (n = 7981) and Year (n = 13); CIs and *p*s from wild cluster bootstrapping with School (subsample n = 2483; full sample n = 2667) and year (n = 13) as cluster variables.

In pre-registered covariate-adjusted analyses, we included population registry data on the pupil’s sex and both parents’ birth country, income, and education. In addition, we ran models that controlled for the proportion of pupils exempted from the tests (or otherwise missing), school’s number of pupils registered for the test, and municipality centrality/rurality by year. These analyses gave a similar pattern of results as the main analyses and are presented in the Supplemental Materials (Tables S2, S3, and S4).

In addition to the 5th grade tests, we pre-specified analyses on 8th grade tests. Unfortunately, data that identified the pupils’ elementary schools (school the previous year) were not available in the registry for 8th grade tests in 2007 and partially missing in subsequent years (see Supplementary Materials, Table S5). An analysis on the available data did not detect any positive effect of the policy and showed tendencies of a negative association between the number of years of free fruit and test scores, B = − 0.17 [− 0.32, − 0.03], *p* = 0.02 for the subsample and B = − 0.07 [− 0.14, 0.01], *p* = 0.08 for the full sample (Supplementary Materials, Tables S6 and S7).

### Exploratory analyses

**Figure 2:**
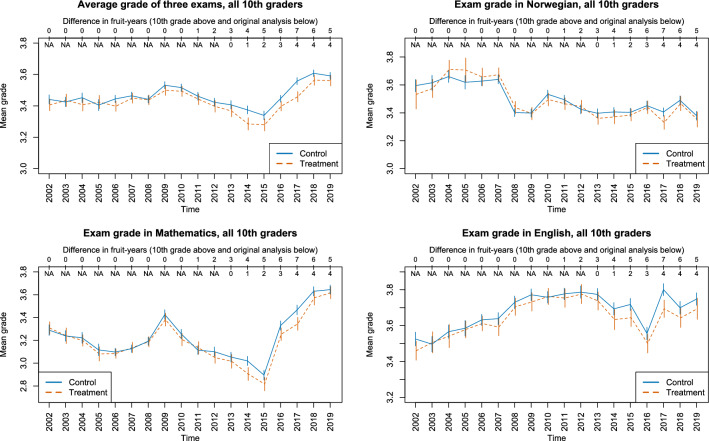
The development in 10th grade exam scores before and during the study period. Error bars are 95% confidence intervals of means. Grade range from 1 (worst) to 6 (best).

To aid the interpretation of the unexpected pattern of results, we acquired additional data on 10^th^ grade exams. The average exam scores for 10th graders in the Treated and Control schools are presented in Fig. 2. The differences in the number of years of exposure to free fruit between the control and treatment groups are presented above the upper horizontal axis. We also indicate the extent the 10th graders were exposed to the policy when they were in 5th grade (below the upper axis) because this makes it easier to see how the data relates to our original analysis. The developments of exam scores pre-policy shows a fairly similar development between treatment and control schools. The trends were parallel, but they appeared to slightly diverge when cohorts were differentially exposed to the free fruit policy. A regression analysis similar to the main analysis suggested negative effects corresponding to a 0.013 decrease in grade points for each year of exposure (see Supplementary Materials, Tables S8 and S9).

In a number of supplementary analyses, we did not detect any positive effects, and we observed estimated effects that were negative. The supplementary analyses included 9th grade national test scores (Table S10), using a single exposure term instead of Phase-in and Phase-out (Table S11), within-municipality analyses (Table S12), and within-subject analyses for a selection of the data where this was possible (Tables S13 and S14, see also Supplemental materials Text S1).

## Discussion

In Norway, during the period 2007–2014, schools with combined elementary and lower secondary education were required to provide one piece of fruit to all pupils every school day. Using ordinary (divided) schools as a control group, we modeled the impact of the policy on test scores. We did not find evidence for a beneficial effect of the policy on academic performance.

In pre-registered analyses, the estimated effects tended to be negative both in a sample of all registered Norwegian 5th graders and in a subgroup known to have a lower intake of fruit and lower academic performance (boys from families with low socioeconomic status). However, in the latter subgroup and in analyses of 8th graders, the statistical support for a negative effect was weak. Explorative analyses on exam data, which used current school type as a proxy for treatment status instead of elementary school type, also indicated a negative development among pupils who were exposed to the policy compared with non-eligible pupils. A number of supplementary analyses either detected no effect or indicated a negative impact.

The free fruit policy was implemented to improve learning outcomes by improving diets^30^. In a survey, a substantial proportion of the personnel responsible for the administration of fruit locally at the schools (e.g., teacher, principal, janitor, or other staff) reported positive perceptions of the program. Specifically, 49% perceived the program to be beneficial for pupils' concentration and 66% for the social environment, while only 5% and 6% did not perceive the program as beneficial for these two aspects, respectively^41^. Some municipalities (i.e., the school owners) have informally reported better concentration and learning outcomes among their pupils during the initial year of the policy, and in a public hearing, several institutions pointed out that such direct beneficial effects are supported by past research and experience^30^. However, scientific literature on the impact of school fruit programs on academic performance is scarce.

Based on the broader literature on school meals, it is not unreasonable to expect a positive effect of food programs on learning. A literature review of studies from developed countries indicates that universal free school meals that included lunch improve academic performance^1^, but results from studies that only studied universal free school breakfast were mixed. Positive outcomes of school breakfast have been reported for concrete behavior observed in the classroom, such as less off-task and out-of-seat behavior^42^, and on cognitive performance, e.g. on memory and attention tasks^43^. A recent US study indicates that academic performance increases when meals are provided by vendors that focus on healthy food, such as whole grain, vegetables, and fruit^2^. Thus, from a general and very broad perspective, one can argue that food provided by the school has the potential to improve academic performance.

### Potential reasons for no positive effect

Why then did the school fruit policy not improve test scores among eligible pupils? The main links in a potential causal chain between the provision of free fruit at school and higher scores on the national tests can be considered as follows: A free fruit policy increases pupils’ exposure to fruits at school. Pupils’ fruit intake at school will then increase, and the pupils’ diet will improve. Improved nutritional status will improve learning, which is assumed to be reflected in higher test scores.

We do not know the exact individual exposure of the free fruit program, but as presented in the introduction, independent studies show that the fruit policy had a positive impact on the pupils’ consumption of fruit^35–37^. This gives us two questions for discussion: Was the impact on diet quality too small? Is the previously shown association between fruit intake and academic achievement in the literature noncausal?

### Low impact on diet quality?

The effect of school meals on cognitive and academic performance has historically been easier to establish in children whose nutritional status is compromised^41,42^. Food insecurity among pupils of low socioeconomic status is associated with a range of behavioral and cognitive problems^44,45^. It could be that school-based nutritional interventions and policies mainly benefit the academic performance of demographic subgroups that experience food insecurity. Some recent US studies on universal free school meals suggest that school meals can improve academic performance across socioeconomic groups^46–48^, but note that the pupils in the US free school meals programs may represent a selective sample, as the program is only available for schools with a high number of pupils defined as poor.

As food insecurity in Norway is rare, but still present^49^, the results from past research may not be generalizable to the current Norwegian context. The diet in Norway, including the children’s diet^39^, is considered to be reasonably good according to the last dietary surveys—although there is room for improvement^50^. The quality of Norwegian diets varies according to socioeconomic status. In Norway, males and individuals of low socioeconomic status eat less fruit than females and individuals of high socioeconomic status^39,51^. Thus, if the provision of fruit is to increase the nutritional status of pupils, this should be more likely in a subgroup of boys with low socioeconomic status (who also have relatively low scores on academic tests and thus greater potential for improvement). Our targeted analyses on this demographic group did not reveal any beneficial effects on test scores, and the estimated negative effects appeared to be of similar magnitude as the effects across all pupils.

### Previous shown associations noncausal?

Several studies have documented associations between diet and academic performance, also specifically between fruit intake and performance^20–22^. The Norwegian free fruit policy has been reported to increased average fruit intake by about 30%^35,36^. Despite these improvements, we observed no positive effects on academic performance. Thus, our results question whether the aforementioned cross-sectional associations reflect a causal relationship. This is also suggested by a recent study that approaches the question of causation using Mendelian randomization^52^. The study reported that different dietary patterns influence performance in various school subjects, but fruit (or vegetables) did not represent a significant component in any of the main patterns found to be beneficial.

In sum, one reason for not finding an positive effect of the policy might be that the association between fruit intake and academic achievement is not causal. Another is that the treatment is too weak, because the provision of free fruit only had a minor impact on the overall diets of the pupils, and/or because the baseline dietary status of Norwegian pupils was already reasonably good^39^. Alternatively, as will be discussed below, the lack of a positive result could be due to side-effects of the policy or methodological issues.

### Potential side-effect of the policy

If we assume that the negative tendency on test scores in the present study truly reflects a causal effect of the policy, it is theoretically difficult to argue that an increased fruit intake decreased academic performance. A more plausible, although speculative, explanation might be that the organization of the program had negative side effects in terms of reduced time for teaching and learning and/or other necessary administration.

The Norwegian school fruit program was criticized in the media for being hasty and disorganized in the beginning^53^. The proposition to repeal the free fruit policy pointed to concerns about how the distribution of fruit was organized (e.g. challenges relating to personnel and logistics)^31^. Most of the persons responsible for the general administration of the fruit at the schools (teacher, principal, janitor, or other staff) reported spending between 10 and 60 min each week^54^. If additionally, the teachers were involved in distributing the fruit to each pupil in class, or if the fruit was provided as a snack that required an extra break, a small disruption or reduction in teaching may be possible. A recent Norwegian qualitative study reported that a newly extended national school-milk subscription scheme added to the teachers’ time burden^55^. Reduced teaching time was also reported in a study on the implementation of a mid-morning breakfast program in Peru^56^. As the direction of our results was unanticipated, we do not wish to emphasize this post hoc explanation.

### Study strengths and limitations

A strength of the study is that the decision to provide fruit to combined schools was related to the administrative status of the schools, not to other characteristics of the schools. However, the school types still differ in several respects (see Table 1). Pupils in the treatment schools have parents with less education and lower income than pupils in the control schools. The treatment schools are located in more rural municipalities, and for 5th grade tests, they scored lower on the tests also in the year before the policy was implemented (see Fig. 1). If these differences reflect stable characteristics of the schools (i.e. the typical demography of the pupils), they are by design accounted for in the analyses. If the composition within schools changes across years or cohorts, this is at least partly accounted for in the covariate-adjusted analyses. However, there may be unmeasured compositional differences that drive the effects. The supplementary within-subject analyses can in principle account for such confounding, yet, we do not consider these analyses as the most trustworthy. Only parts of the data could be used, there were few clusters to account for potential heterogeneity in change scores, and the analyses assume equal influence of fruit in different phases and at different ages. The within-subject analyses detected no beneficial effect of the policy on learning and tended more towards negative than positive estimates.

The present analyses are like intention-to-treat analyses, where we use data from all pupils regardless of fruit consumption. In other words, we estimate the effects of a policy, not directly the effects of eating fruit. Relatedly, we do not have school details regarding other relevant programs running before, during, or after the free fruit policy. Few elementary schools have school meal programs in Norway, but the number is increasing. In 2013 approximately half of the combined schools, but only one-tenth of the ordinary elementary schools offered paid services such as school canteen, most of the schools offer subscription-based (paid by the parents) milk and other beverages, and 57% of the ordinary elementary school leaders reported to have a kind of fruit arrangement (mostly a subscription program)^57^. However, due to low participation, fruit subscription has a limited impact compared to a free fruit program^58^. For example, in 2006, just before the implementation of the free fruit policy, 41% of eligible schools participated in a national subscription program, and 28% of pupils at participating schools subscribed, reaching only 12% of potential pupils^59^. As the subscription-based fruit programs were available to all schools before the free fruit policy, to the ordinary elementary schools during the time of the policy, and again to all schools after the abolishment, this is in principle not a problem for identifying the effect of the free fruit policy. Furthermore, the pupils that subscribed were typically of high socioeconomic status and consumed more fruit and vegetables in the first place compared with non-subscribers^58^.

### Implications

The surprising results of the present study make it difficult to assess potential implications. While the results cannot be taken as evidence against the benefits of eating fruit, they do suggest that the mere provision of free fruit does not automatically translate into improved academic outcomes. Policymakers and educational institutions should exercise caution when anticipating educational benefits from nutritional programs. Nutritional programs may be sensitive to context; for instance, they may be ineffective in settings where food insecurity is relatively rare. Our results do not address the possibility of satisfaction from receiving free fruit or potential health benefits from consumption. However, they do suggest that such potential benefits are unlikely to include improvements in academic performance in a highly developed country with reasonable good dietary habits. As unintended consequences of operational aspects may offset potential benefits, future policy development should place greater emphasis on the administrative burden and time allocation at the local level.

## Conclusion

In Norway, a Western country with low food insecurity but a lower-than-recommended fruit and vegetable intake, the government required some school types to give pupils one piece of free fruit every school day. Although informal evaluations (e.g., satisfaction and perceptions of pupils’ concentration) have been generally positive, analyses on register data in the present study reveal no or even a negative effect of the policy on academic performance.

## Methods

### Participants and data

The full datasets on national tests (Mathematics, English, and Reading) consisted of all registered pupils in Norway in 5th and 8th grade in the years 2007–2019 (790,242 pupils in 5th grade and 798,869 in 8th grade) and all pupils in 9th grade in the years 2010–2019 (only Mathematics and Reading). Unless individual arrangements are made for early or late school entry, pupils are typically 9 or 10 years old at the time of the national tests in 5th grade, 12 or 13 years old at the time of the 8th-grade tests, and 13 or 14 years old at the time of the 9th-grade tests. Characteristics of the sample used in the main analysis are presented in Table 1 and characteristics of the 8th grade sample are presented in the Supplementary Materials, Table S5.

Data on exam scores for exploratory analyses were obtained at the level of schools and included data from 954 schools. This covered approximately 90% of Norwegian pupils. The data were results from exams in Mathematics, English, and Norwegian in the years 2002–2019. The exams are given in 10th grade at the end of the school year when the pupils are 14 or 15 years old.

### Context and study design

The free fruit policy was in effect from the school year that started in autumn 2007 (the school year 2007/2008) to the end of the school year that started in autumn 2013 (the school year 2013/2014). The policy included all schools with lower secondary education, which also meant that elementary pupils in combined elementary and lower secondary schools (20% of elementary pupils in Norway) were covered. Children in ordinary elementary schools (80%) were not eligible for free fruit and is used as a control group.

The policy was in effect for a duration of 7 years, leading to a graded exposure to free fruit for pupils in the combined schools. Specifically, the number of years each cohort was exposed to free fruit increased after the policy’s introduction and subsequently decreased following its abolition. During lower secondary education (8th–10th grade) either all pupils (in the school years 2007/08–2013/14) or none (in the school years before 2007/08 and after 2014/15) received free fruit in the year before testing. There is a tradition for bringing packed lunch in Norway, and school-provided breakfast or lunch is not common in elementary school. This means that for the majority of the pupils eligible for free fruit, the piece of fruit was the only food item provided by the school.

The study consists of repeated cross-sections of pupils, which provides school-level longitudinal data. We compare the developments in the treatment group with the developments in the control group.

### Categorization of treatment status

Schools were categorized into treatment and control according to the maximum and minimum grade levels, which indicated whether the schools were combined 1st–10th grade schools. Schools with a maximum grade level above 8th grade were defined as combined (treated) and schools with maximum grade level below 8th grade were defined as ordinary elementary schools (control). Current school were registered for all tests and for the 8th grade tests the previous school (i.e., elementary school) was registered. For the 9th and 10th grade tests, we did not have access to the pupil’s elementary school, and we therefore used their lower secondary school status as a proxy for elementary school status. That is, pupils who were registered at combined schools (lowest grade = 1st) were coded as treated, and pupils registered at standalone lower secondary schools (lowest grade = 8th) served as control. To see if current school in lower secondary education was a good proxy for a pupil’s school type in elementary school, we compared current and previous school types in 8th grade (for which both were recorded). Approximately 88% of the students were recorded with the same school status (combined versus divided) for previous and current school.

### Targeted analysis of boys with low socio-economic status

Boys eat less fruit and perform worse on national tests than girls, and children from families with lower socioeconomic status eat less fruit and score lower than average on academic tests^39,40^. Thus, boys from families with low socioeconomic status represent a group of particular interest that has upward potential both nutritionally and in terms of academic performance. Some groups of immigrant origin have a low consumption of fruit, while the traditional food of other groups includes a high amount of fruits and vegetables^60^. To focus on a more homogeneous group that also avoids the issue of potential demographic changes among immigrants, we decided to include only non-immigrant boys in the targeted analysis. Specifically, the criteria for inclusion in this targeted analysis were: (a) male, (b) non-immigrant status according to categories by Statistics Norway, (c) member of a household with income below the yearly median, and (d) neither parent registered with completed higher education.

### Permissions and waiver of consent

The study was approved by the Data Protection Officer at the Norwegian Institute of Public Health. All methods were carried out in accordance with relevant guidelines and regulations. Informed consent was not required due to §8 in the Personal Data Act.

### Deviation from the pre-registration

Analyses were pre-registered: https://osf.io/uefjp. Due to delays in the project, we received an additional year of data (i.e., 2019). Original pre-registered analyses without the extra year are included in the Supplemental Materials (Table S15). We originally planned to rely on the inferential statistics from Linear Mixed Models because these models provided more conservative results than cluster-robust Fixed Effects models in simulations (see pre-registration). However, due to non-convergence and modeling issues (heterogeneity and difficulties in analyzing standardized scores due to singular fit and non-convergence), we focus on the cluster-robust Fixed Effects models. The inferential statistics of the Linear Mixed Models were not more conservative (Supplemental Materials, Text S2 and Tables S16 and S17).

We specified three different impact models (see Supplemental Materials, Table S18). The first impact model was our a priori best guess that the policy would produce a diminishing impact for every additional year of free fruit received (1 year coded 1, 2 years coded 1.5, etc.), based on the idea that free fruit could establish better habits during initial years or that a potential beneficial effect would be due to the elimination of some nutritional deficit. In the absence of such positive effects, the diminishing impact model is best considered as an arbitrary scale of exposure, and its coefficients are not directly interpretable. Therefore, we included this model only in the supplementary materials (the results were consistent with our second impact model). We focused on our second impact model, which was pre-specified as a model to interpret the magnitude of potential effects. This impact model was based on the actual number of years of free fruit (1 year = 1, 2 years = 2, etc.). We additionally report results from a third pre-registered impact model that used any fruit versus no fruit (either exposure or not; none = 0, 1 year = 1, 2 years = 1, etc.), but this model could not be used for the original 8th grade analysis due to missing data in the first year of the study period (when exposure was 0). In the pre-registration, we included specifications for Bayes Factors, but for improved readability we chose to omit the reporting of these in the main text. Bayes Factors are reported in the Supplemental Materials (Text S3, Table S19).

### Statistical analyses

The main analyses were regression analyses that estimated the development in the Treatment group’s test scores as a function of exposure to the free fruit policy, relative to the scores of the non-exposed control group during the same years. The exposure to the fruit policy was estimated by separate Phase-in and Phase-out terms. Note that this did not reflect a gradual roll-out of the policy, but instead a gradual accumulation and decrease in the number of years the pupils were exposed to the policy. We additionally included a control variable for whether students were eligible for free fruit at the time of the test. Given that the test is administered early in the semester, this variable controls for the potential effect of short-term exposure to the policy (see supplemental materials, Table S1). A priori, we chose to focus on the Phase-in term (See pre-registration https://osf.io/uefjp).

The main analyses included fixed effects of year and school by test type (Mathematics, English, Reading). The Fixed Effects regressions were estimated by OLS with the user-written function ‘reghdfe’^61^ in Stata 15.0. Potential heteroscedasticity and dependence within time and school clusters were accounted for by two-way cluster-robust inference. The inferential statistics were either based on the default cluster-robust standard errors or wild cluster bootstrapping (999 replications; null imposed) with the user-written function ‘boottest’^62^, as specified in the tables.

To illustrate the variation in test scores over time, and for communicative purposes, we chose to focus on the original scores of the tests, but we report the main results of standardized variables (M = 0 and SD = 1 within each test type by year) in text and further results on standardized scores are referred to in text and included in the Supplemental Materials. The standardized scores were standardized according to year and test type (English, Reading, Mathematics). That is, the mean of all observations (year by test type) was subtracted from the original scores, and the differences were divided by the standard deviation (year by test type).

### Supplementary Information

Supplementary Information.

## Data Availability

The data is not publicly available but may be obtained by application to Statistics Norway (only for researchers affiliated with approved research institutions). See https://www.ssb.no/en/omssb/tjenester-og-verktoy/data-til-forskning.
